# Height of Driver’s Eye and Taillight Measurements from a Camera-Based Roadside Setup

**DOI:** 10.3390/s19071611

**Published:** 2019-04-03

**Authors:** Yohan Dupuis, Azzedine Chabani, David Doucet, Peggy Subirats

**Affiliations:** 1 Normandie Univ, UNIROUEN, ESIGELEC, IRSEEM, 76000 Rouen, France; 2 Cerema Normandie-Centre, 10 Chemin de la Poudrière, 76120 Le Grand-Quevilly, France; azzedine.chabani@cerema.fr (A.C.); david.doucet@cerema.fr (D.D.); peggy.subirats@cerema.fr (P.S.)

**Keywords:** camera, height, eye, taillight

## Abstract

This paper presents an approach that can be used to measure height of driver’s eyes and rear position lamps from a video, i.e., two important metrics used to set sight distance standards. This data plays an important role in the definition of geometric design of highways and streets. Our method automatically estimates the camera pose with respect to the road. It then requires selecting two points to obtain the height. New vehicles tend to be higher and larger. Consequently, this information shoud be updated. This approach has been applied on a large panel of vehicles. Our method was evaluated on vehicle height measurements. Our results suggest that our method achieves less than 1.8 cm (0.7 in) mean absolute error. Our experiments show an increase in the height of driver’s eyes and taillights.

## 1. Introduction

Sight distances play an important role in road design. In fact, road design must help vehicles to stop safely, given breaking capacities, as soon as the driver sees a vehicle stopping in the same lane.

Passing maneuvers are another example of sight distance applications in road design. When passing is allowed, the road design must ensure that the driver can have enough space to complete a passing maneuver given the available sight distance.

These constrains are also applicable to autonomous or automated vehicles. In fact, given its sensing capabilities, the vehicle must run smoothly, perform overtaking safely or be able to stop if a car stops in its current lane. Sight distances have impact on vertical and horizontal alignments of road elements [1]. It influences horizontal curve design, crest vertical curve design or road grades.

Sight distances are influenced by the height of scene perception sensors, i.e., a LiDAR (Light Detection And Ranging), a camera or the driver eye. Today’s standards use the average height of driver eyes. For instance, the 2011 edition of AASHTO (American Association of State Highway and Transportation Officials) Greenbook uses height of the driver’s eye equal to 1.08 m (3.54 ft) [2] based on Fambro study [3]. It was 1.65 m (5.41 ft), 1.15 m (3.77 ft), 1.07 m (3.51 ft) in 1920, 1965 and 2000 editions, respectively. The height of the driver eye used in the UK is 1.05 m (3.45 ft) and relies on [4].

The height of the target also has an impact on sight distances. Headlights or taillights are often considered as targets for sight distance computations. The 2011 AASHTO Geometric design guide [2] suggests an object height of 600 mm (2.0 ft) for stopping and decision sight distances and 1080 mm (3.55 ft) for passing and intersection sight distances The height of object height in the UK ranges from 0.25 m (0.82 ft) to 2.0 m (6.56 ft).

For the time being, French regulations use a height of a driver’s eye of 1 m (3.28 ft) and the height of target objects of 0.6 m (1.97 ft) [5]). They were set in 2000. Both heights were defined based on car manufacturer data sheets and a survey carried out at a toll station close to Paris. The height of target objects of 0.6 m was consistent between both sources of information. The height of target objects corresponds to the 10th percentile of the height distribution of the rear tail light lower limit of the emitting area. The survey carried out at the toll station did not allow to accurately measure the height of the driver’s eye. As a result, the Fambro study [3] was used to define the height of driver’s eye. The height of 1 m was chosen as it represents the 2.5th percentile.

Few studies have investigated how to obtain and update the height of driver eyes standards. Approaches can be empirical. In [3], Fambro et al. used a color camera. They placed markers on the road to be used as a scale and drove vehicles with known heights for calibration purposes. In 2012, Reference [6] proposed to use the car model to find out the height of driver eyes. The camera is placed in a parallel fashion with the side plane of the vehicle. The vehicle height is used to scale the driver’s eye height measurement. Methods can also be analytical. In [7], the researchers used a two-step approach. First, the Society of Automotive Engineering (SAE) standard on seating reference point (SgRP) is used. Then, the UMTRI (University of Michigan Transportation Research Institute) eyellipse model, based on the observations of 68 drivers collected in a lab environment, is used in conjunction with the SgRP to obtain eye height distribution.

Our work addresses the problem of empirical height measurements from camera-based temporary roadside measurements. We aim at proposing a framework that is versatile enough to measure the height from one camera but could also provide other information such as the lateral location of the vehicle on the road if necessary. It must be robust and requires high repeatability. The process is automatic except for the selection of the point required to find the object of interest’s height. It will have to locate 3D points in the scene.

Measuring the height from the location of 3D points in the scene requires to obtain the extrinsic calibration, i.e., the location of the camera with respect to the ground plane and the traffic direction. As mentioned in [8], calibration patterns do not naturally exist in traffic scenes. Consequently, there is a growing interest in automatic calibration from CCTV cameras. Reference [9] relies on vanishing points to find out the camera orientation with respect to the ground plane. The traffic’s main direction and gradients from the vehicles are used to find the vanishing points.

Zheng and Peng go a step further as they estimate both intrinsic and extrinsic parameters [8]. They also relied on vanishing points to estimate the camera orientation. Several approaches are proposed to recover the camera translation with respect to the road plane origin. Overall, CCTVs are placed far from the ground [10]. Consequently, the camera field of view covers a large area. The car trajectory can be observed for a long period. Moreover, the three main directions from cuboid-like car shape can easily be seen.

**Figure 1:**
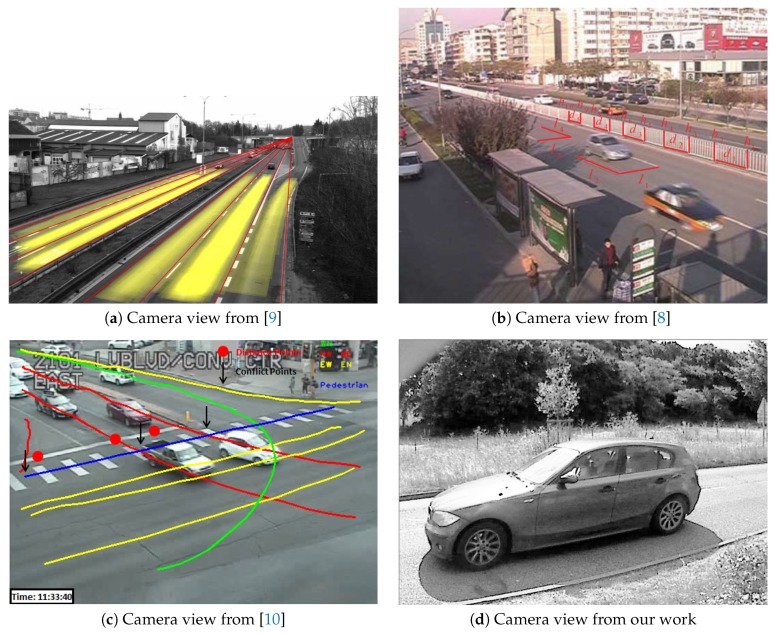
Camera views used for extrinsic calibration.

As it can be seen in Figure 1d and Figure 4b, measurements from ground level and temporary setups are not so convenient. In fact, high posts may not frequently exist in the environment. Moreover, regulation requires using guy-wire for temporary high posts. A larger space is required for guy-wire that might not be available at the measurement location.

Consequently, our work proposes a framework that allows to measure heights in traffic scenes from ground level cameras. Our method does not require the traffic to be stopped. Our approach is suitable for temporary setups in any road environments (urban, road side, ...) and works both in daytime or low illumination situations.

The paper is organized as follows. Section 2 presents a flexible framework that allows to locate 3D points on the car. Section 3 first qualifies the performance of our approach on the vehicle height task and presents the height of driver eyes and taillight distribution. Section 4 concludes the paper.

## 2. Methodology

A camera is a sensor that images the world with the help of a lens and an image sensor. The image is a perspective projection of the world. The camera model relates a 3D point and its 2D projection as follows:

(1) $$\lambda \left[\begin{array}{c} u\\ v\\ 1 \end{array}\right]=K\left[\begin{array}{cc} R & t \end{array}\right]\left[\begin{array}{c} x\\ y\\ z\\ 1 \end{array}\right]$$

where:

$P={\left[\begin{array}{cccc} x & y & z & 1 \end{array}\right]}^{T}$ is a 3D point in the World frame.
$p={\left[\begin{array}{ccc} u & v & 1 \end{array}\right]}^{T}$ is the projection of P in the image.
λ is a scaling factor.
$\left[\begin{array}{cc} R & t \end{array}\right]$ is the transformation that brings point from the world coordinate system to the camera coordinate system. *R* and *t* are also known as extrinsic parameters.
*K* is the transformation that brings point from the camera coordinate system to the image frame.

*K* embeds the intrinsic parameters of the camera:

(2) $$K=\left[\begin{array}{ccc} rf & s & u_{0}\\ 0 & f & v_{0}\\ 0 & 0 & 1 \end{array}\right]$$

where:

$\left(u_{0},v_{0}\right)$ is the coordinate of the principal point in the image frame
*f* is the focal length
*r* is the aspect ratio.
*s* is the skew factor.

*s* and *r* are considered respectively equal to 0 and 1 [11].

Equation (1) is applicable to perfect cameras. For real cameras, the lens quality and misalignment between the lens and the image sensor may introduce deformations in the resulting image called distortions. Consequently, Equation (1) must be modified as follows:

(3) $$\lambda \left[\begin{array}{c} u\\ v\\ 1 \end{array}\right]=Kd\left(\left[\begin{array}{cc} R & t \end{array}\right]\left[\begin{array}{c} x\\ y\\ z\\ 1 \end{array}\right]\right)$$

where:

*d* is a nonlinear transformation parameterized by the distortion coefficients.

The intrinsic parameters as well as the distortion coefficients can be recovered with a camera calibration. Standard camera calibration approaches include works from [12] and [13].

Measuring the height from an image from 3D information requires to measure the distance from the ground to the point of interest. Consequently, the intrinsic and extrinsic parameters must be known as well as the scaling factor. Unfortunately, the scaling factor is lost due to the projective property of the transformation.

The best that can be recovered from a pixel in an image is a 3D line that passes through the origin of the camera coordinate system and the back projection of the pixel on the unit plane.

**Figure 2:**
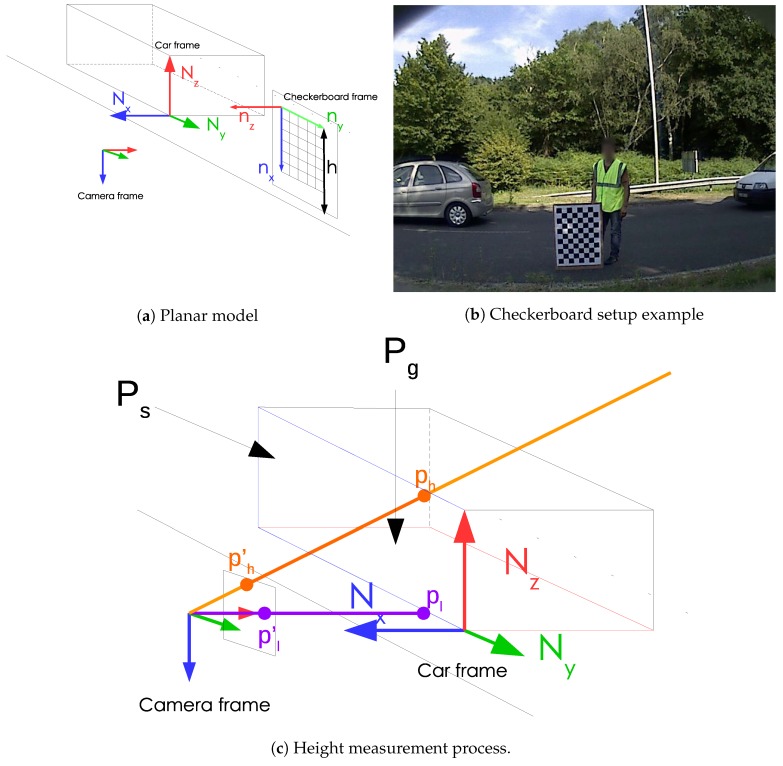
Extrinsic calibration and height measurement.

In the example given in Figure 2c, *p* cannot be recovered. However, only the line (black line) passes through *p* and the camera’s coordinate system origin can be recovered as follows:

(4) $$p=d^{-1}\left(K^{-1}\;\cdot \;P\right)$$

Still, we do not know how far the 3D point is on the line from a single image, Consequently, in order to recover object heights from a single image, either several cameras must be used or a single camera with a-priori information. As it can be seen in Figure 2, the road can be approximated as a plane defined by the normal $N_{z}$. A vehicle can be represented by a cuboid. We consider that the vehicle vertical axis is parallel to the road plane normal. In order to measure the height, we must find the location of the car frame origin with respect to the camera frame. The car can be anywhere on the road platform. However, car motions are mainly in the same directions, i.e., the longitudinal direction.

In order to obtain a method that does not require knowing the car model, we applied a three-step approach. The key information are the directions of $N_{x}$, $N_{y}$ and $N_{z}$ in the camera frame as well as the location of the ground plane. One could try to detect the vehicle edges in order to find these parameters. However, with today’s car designs, it would be highly inaccurate to try to estimate the direction from the roof or the door edges. Moreover, the location of the car frame origin is still missing.

In order to obtain robust and accurate estimations of $N_{x}$, $N_{y}$ and $N_{z}$, we employ a checkerboard.

The checkerboard is placed vertically on the road platform as shown in Figure 2a. As it can be seen $N_{x}$, $N_{y}$ and $N_{z}$ are respectively parallel to $n_{z}$, $n_{y}$ and $n_{x}$. The checkerboard origin is located at the top-left corner of the checkerboard.

First, we must find the location of the checkerboard frame in the camera frame. In other words, the transformation that relates the camera frame and the checkerboard frame must be found. Checkerboards are classic patterns used in camera calibration processes. Standard calibration techniques estimate the intrinsic parameters from several views of a checkerboard patterns as well as the extrinsic calibration, i.e., the location and orientation of each checkerboards in the camera frame.

In order to obtain good results, it is better to have a checkerboard that covers a large part of the image. Moreover, it is highly advised to have different orientations of the checkerboard to achieve robust intrinsic parameter estimation. In our case, the calibration is performed under traffic. As a result, only small time-windows are available to place the checkerboard. We favored one good pose of the checkerboard that we record over a small amount of time (up to 5 s). This single pose will result in a poor calibration. It will be even worst because the checkerboard size in the image would be rather small.

All things considered, we first calibrate the camera in a lab. Consequently, the intrinsic parameters are accurately estimated with a retro-projection error of less than one pixel despite lens distortions. Still, we must estimate the pose of the checkerboard in 3D from its projection in the image, i.e., 2D points.

**Figure 3:**
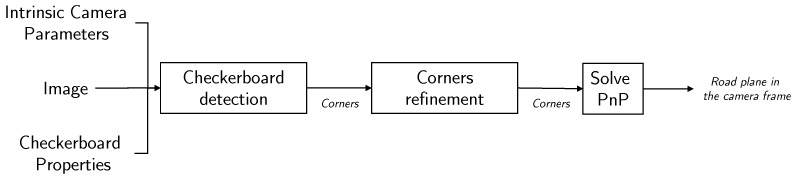
Road plane estimation pipeline.

This problem is known as the perspective-n-point proble m (PnP problem). PnP solving require at least three-point correspondences. It is a well-established problem. Several approaches can be found in literature from the computer vision community [14,15,16,17]. Consequently, the rotation matrix R and translation vector t from the camera frame to the checkerboard frame are obtained by solving the PnP problem. The pipeline summary is presented in Figure 3.

It allows to find the directions $n_{i}$ as follows:

(5) $$n_{x}=\left[\begin{array}{cc} R & t \end{array}\right]{\left[\begin{array}{ccc} 1 & 0 & 0 \end{array}\right]}^{T}$$

(6) $$n_{y}=\left[\begin{array}{cc} R & t \end{array}\right]{\left[\begin{array}{ccc} 0 & 1 & 0 \end{array}\right]}^{T}$$

(7) $$n_{z}=\left[\begin{array}{cc} R & t \end{array}\right]{\left[\begin{array}{ccc} 0 & 0 & 1 \end{array}\right]}^{T}$$

As the checkerboard frame origin is not located at the ground level, the coordinate of the ground point is given as:

(8) $$X_{g}=t+h\times n_{x}$$

where:

*h* is the height in meter of the checkerboard origin from the ground.

3D planes are geometrical object algebraically defined by a 1 × 4 vector P = $\left[\begin{array}{cccc} a & b & c & d \end{array}\right]$. A 3D point X = $\left(x,y,z\right)$ belonging to the plane must fulfill:

(9) $$\left[\begin{array}{cccc} a & b & c & d \end{array}\right]{\left[\begin{array}{cccc} x & y & z & 1 \end{array}\right]}^{T}=0$$

where:

$\left[\begin{array}{ccc} a & b & c \end{array}\right]$ corresponds to the normal direction of the plane. *d* force the true location of the plane.

Thanks to our PnP solution, the ground plane normal is already known. In fact, as it can be seen in Figure 2, $n_{x}$ and $N_{z}$ are parallel. As a result, $P_{g}$ is equal to ${\left[\begin{array}{cc} -n_{x} & d \end{array}\right]}^{T}$. *d* can be easily obtained as it must solve:

(10) $$\left[\begin{array}{cc} -n_{x} & d \end{array}\right]{\left[\begin{array}{cc} X_{g} & 1 \end{array}\right]}^{T}=0$$

(11) $$d=n_{x}^{T}\;\cdot \;X_{g}$$

We are consequently able to recover the ground plane definition in the camera frame with:

(12) $$P_{g}=\left[\begin{array}{cc} -n_{x} & n_{x}^{T}\;\cdot \;X_{g} \end{array}\right]$$

We have gathered all the information required to start the measurements. As it can be seen in Figure 2c, $N_{x}$ is parallel to $n_{z}$ and $N_{y}$ to $n_{y}$.

Figure 2c explains how we recover the 3D height from 2D image points. First, a pixel as close as possible to the contact of the vehicle wheel with the ground, represented as $p_{l}$, must be chosen. It defines $p_{l}^{\prime }$, the normalize point coordinate of the selected pixel. A second pixel corresponding to the object projection from which we want to know the height, represented as $p_{h}$, must be chosen resulting in $p_{h}^{\prime }$ once in normalized coordinates.

$p_{h}^{\prime }$ and $p_{l}^{\prime }$ actually represents two 3D directions represented as orange and purple lines respectively. We first use $p_{l}^{\prime }$ to find the location of the vehicle side plane. We have to find the intersection of the purple line with the ground plane found previously. $p_{l}$ is computed as follows:

$$p_{l}=\frac{n_{x}^{T}\;\cdot \;X_{g}}{n_{x}^{T}\;\cdot \;p_{l}^{\prime }}\;\cdot \;p_{l}^{\prime }$$

$p_{l}$ allows to find the lateral position of the vehicle side plane $P_{s}$. As done previously with $X_{g}$, we use the normal directions found with the checkerboard. The vehicle side plane definition is given by:

(13) $$\left[\begin{array}{cc} C_{x}^{T} & d \end{array}\right]{\left[\begin{array}{cc} p_{l}^{T} & 1 \end{array}\right]}^{T}=0$$

(14) $$\left[\begin{array}{cc} n_{z}^{T} & d \end{array}\right]{\left[\begin{array}{cc} p_{l}^{T} & 1 \end{array}\right]}^{T}=0$$

(15) $$d=n_{z}^{T}\;\cdot \;p_{l}$$

We are consequently able to recover the side plane $P_{s}$ definition in the camera frame with:

(16) $$P_{s}=\left[\begin{array}{cc} -n_{z}^{T} & n_{z}^{T}\;\cdot \;p_{l} \end{array}\right]$$

Finally, we use $p_{h}^{\prime }$ to recover $p_{h}$ as follows:

(17) $$p_{h}=\frac{n_{z}^{T}\;\cdot \;p_{l}^{\prime }}{n_{x}^{T}\;\cdot \;p_{l}^{\prime }}\;\cdot \;p_{h}^{\prime }$$

$p_{h}$ may not be vertically aligned with $p_{l}$. As a result, we considered the height of $p_{h}$ as the distance of $p_{h}$ to the ground plane $P_{g}$:

(18) $$h=\frac{p_{h}^{T}\;\cdot \;P_{g}}{-n_{x}}$$

## 3. Results

### 3.1. Experimental Setup

Our method requires a camera and a checkerboard. In our experiments, we used an Axis 211W camera. It uses the 1/4 Progressive scan RGB CMOS VGA image sensor. The varifocal lens provided with the camera was disabled and set to 3 mm in order to obtain the largest FOV (i.e., 67°). Its F is equal to 1.0. The focal length as well as the distortions are estimated during the intrinsic camera calibration and used during the road plane estimation and heigth measurement pipelines. It outputs 640 × 480 images at a frame rate of 30 frames per second. The camera was set to its maximum fps in order to reduce motion blur. The exposure time cannot be set on the camera. Recordings were performed during both day and low light experiments as required by French standards. Low light experiments correspond to recording at nighttime with street light nearby.

**Figure 4:**
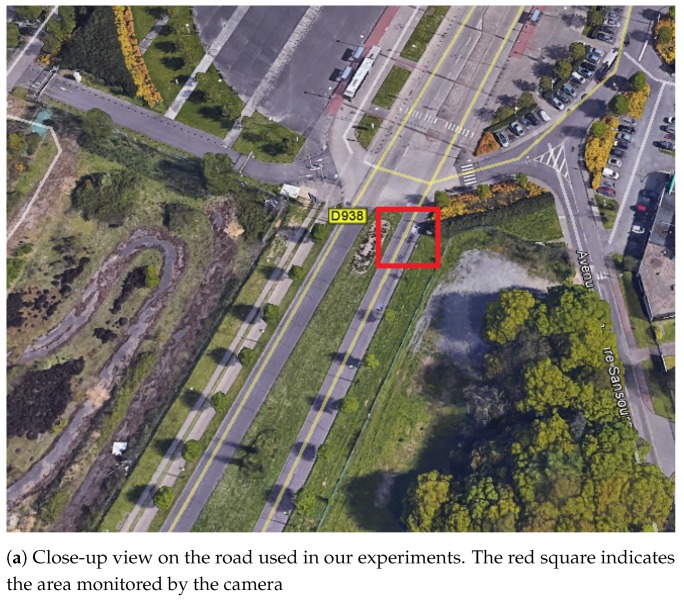

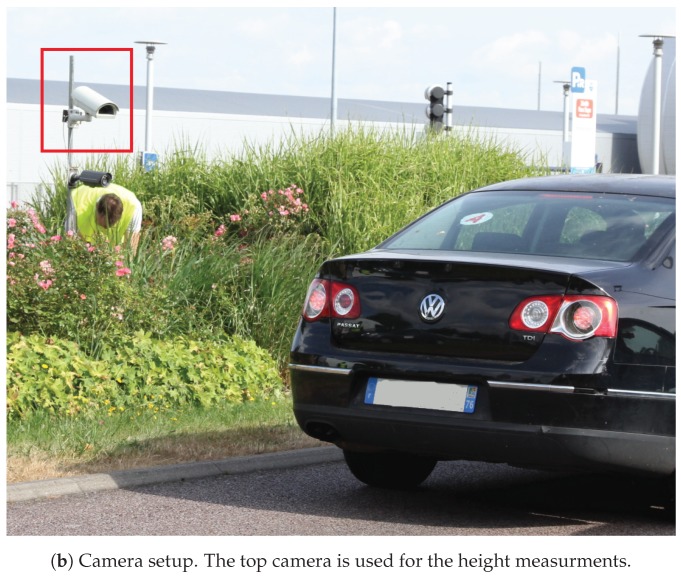
Camera installation used in the experiments.

The camera and recording set were placed on a post at 1.5 m (4.92 ft) high (see Figure 4). The road monitored corresponds to a divided highway used by commuter from the South of Rouen area (see Figure 4).

The checkerboard is printed in a A0 format. It is composed of 5 × 7 squares and an area of interest of 50 cm × 70 cm (19.68 in × 27.55 in). The OpenCV library was used to calibrate the camera, detect the checkerboard and solve the PnP problem. The camera extrinsic calibration with the road is performed under traffic. It requires less than 5 s of the A0 checkerboard images to obtain an accurate estimation of the extrinsic calibration.

### 3.2. Dataset

**Figure 5:**
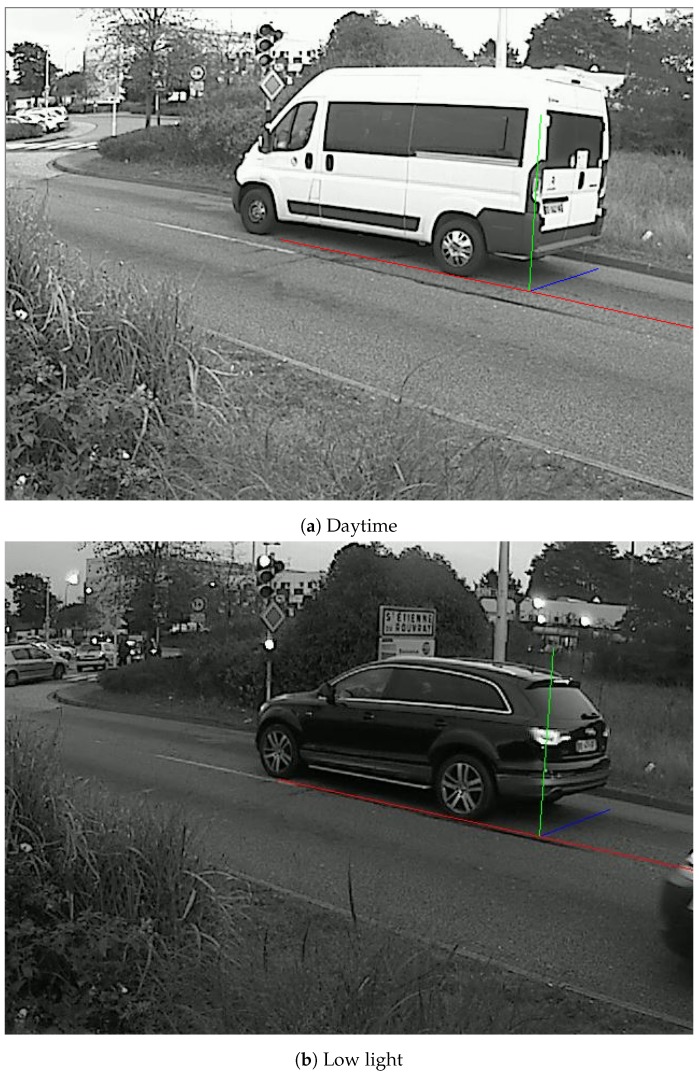
Samples from the dataset. Red line: projection of the longitudinal direction, Green line: projection of the vertical direction, Blue line: projection of the transversal direction.

Our work includes two datasets. The first dataset includes 400 vehicles in daytime. The second dataset is based on 250 vehicles captured with low light. (c.f. Figure 5). Each captured vehicle was individually identified with its car brand, car model and estimated year of manufacturing in order to deal with vehicle design updates over years. Then, the corresponding car manufacturer datasheets were browsed to find the corresponding heights. These heights are considered as the ground truth for each vehicle.The vehicle height measured with our approach is then compared with the ground truth in order to assess the height measurement error of our process. Still, this height provided by car manufacturers can be affected either by the vehicle load or dynamics. The vehicle load will slightly reduce the vehicle height. Braking can slightly increase the taillight height. Uncertainty regarding the ground truth could be considered as equal to 3 cm. Vehicles are located 1.5 m (4.92 ft) to 5 m (16.4 ft) away from the camera.

### 3.3. Extrinsic Calibration Result

Our experiments were performed under trafic in outdoor environments. Consequently, no direct ground truth is available regarding the camera pose with respect to the road. We propose to analyse how the road frame is projected on examples from our sets.

Figure 5 shows two samples. The images display the result of the extrinsic calibration with the help of the estimated directions.

A closer look at the red lines, i.e., the longitudinal ground level direction, shows that it passes through both tire-ground contact location. The green line gives the vertical direction. It is also coherent as it fits the rear bumper and the top left corner of the vehicle roof.

It shows that our extrinsic calibration is correct despite strong image distortions and observations on two different lanes.

### 3.4. Performance Evaluation

The height measurement performance is assessed with respect to the height provided by the car manufacturer as it is the only ground truth available. Measured and ground truth heights are provided in millimeters. Still, driver’s eye and tailight heights in highway design standards are expressed in centimeters. In accordance with the standards, measured and ground truth heights were converted to centimeters and rounded to the closest integer. Points $p_{h}^{\prime }$ and $p_{l}^{\prime }$ (c.f. Figure 2c) required to obtain the height are manually selected in the image. In fact, the robustness and repeatabilty required to set standards does not allow to use automatic detection.

Table 1 presents several performance metrics. The mean absolute error (MAE) is less than 2 cm (0.78 in) when considering daytime and low light conditions. The performance is better for low light conditions. As it can be seen in Figure 5, contrasts are better on the low light view. Consequently, it is easier to select the ground point as well as the point of interest. The root mean squared error is really close to the MAE. This is due to a really small standard deviation.

**Figure 6:**
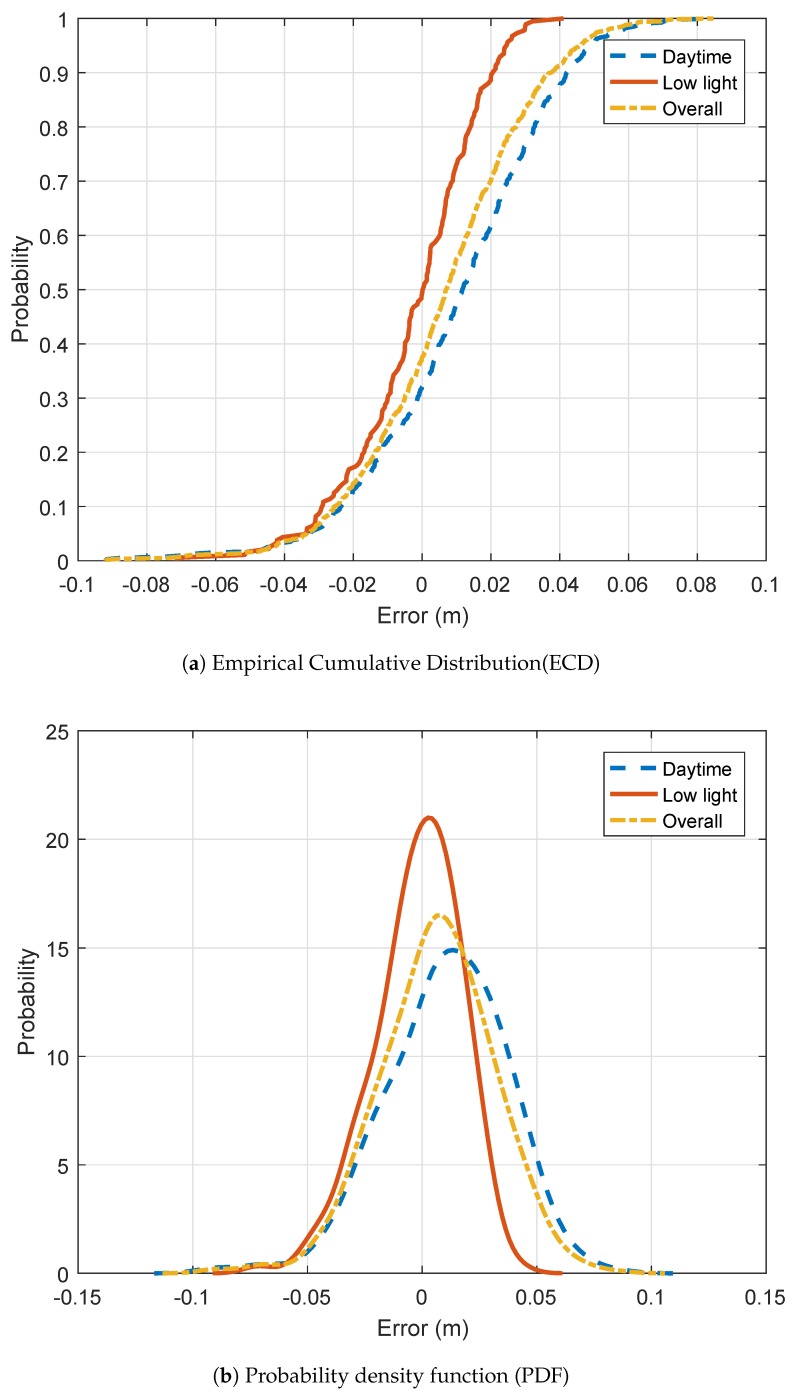
Absolute error distributions.

Figure 6a focuses on the distribution of the absolute error. Overall, it can be seen that less than 10% of the samples have an error smaller than 2 cm (0.78 in). The distribution is biases toward 2 cm (0.78 in). Less than 10% have an error larger than 4 cm (1.57 in). The resulting specifications are even better for the low light conditions where 95% of the samples have an error less than 3 cm (1.18 in). As explained earlier, better contrasts lead to a finer selection of the point of interests.

Figure 6b shows the distribution of the relative error. Experiments with low light lead to errors lower than 3%. 60% of the nighttime samples have errors lower than 1 cm (0.39 in). The daytime dataset results in errors lower than 5%. 60% of the samples have errors less than 1.5 cm (0.59 in). The shape of both daytime and low light ECDs looks similar except at the left-hand side of the distribution where the daytime curve first flatten and then increase again.

**Figure 7:**
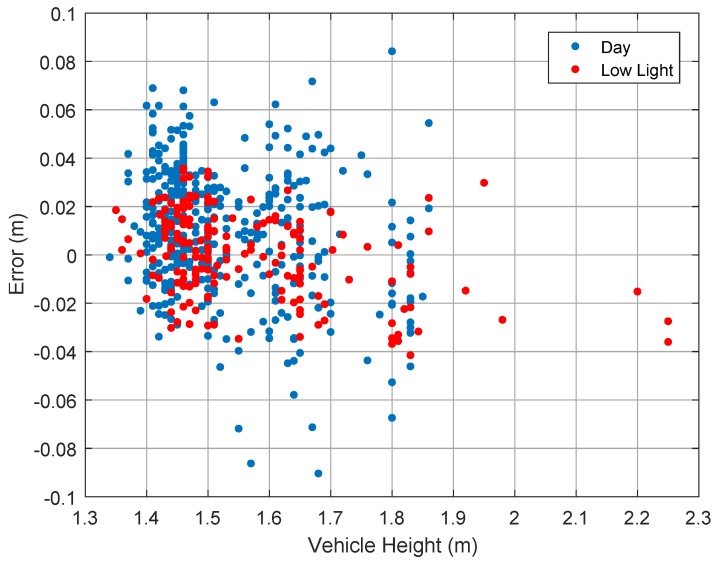
Signed error as function of vehicle height in meter.

In Figure 7, we investigate the correlation of the absolute error and the true vehicle height. It can be seen that some correlation exists between the vehicle height and the signed error. The absolute error is negatively correlated with the vehicle height. In other words, the height is slightly overestimated for small vehicles and underestimated for large vehicles. The correlation is less significant for daytime samples.

A closer look at absolute values of absolute error gives correlation coefficients of 0.06 and 0.47 for daytime and low light respectively. As a result, the approximation in the point of interest selection totally mitigates a likely extrinsic calibration error. Still, the low light dataset reveals that the error tends to increase with the vehicle size.

This might be caused by a slight error in the estimation of the side plane orientation. The plane might be slightly outward of its real orientation. This might be caused by several factors: camera intrinsic calibration, calibration plane orthogonality with the real orthogonal direction with respect to the road surface and a too strong approximation of the road shape and car shape. Still, this can be mitigated as the MAE is less than 1.5 cm (0.59 in), 95% of the samples have an error smaller than 3 cm and all samples have an error less than 3%.

### 3.5. Height of a Driver’s Eye

**Figure 8:**
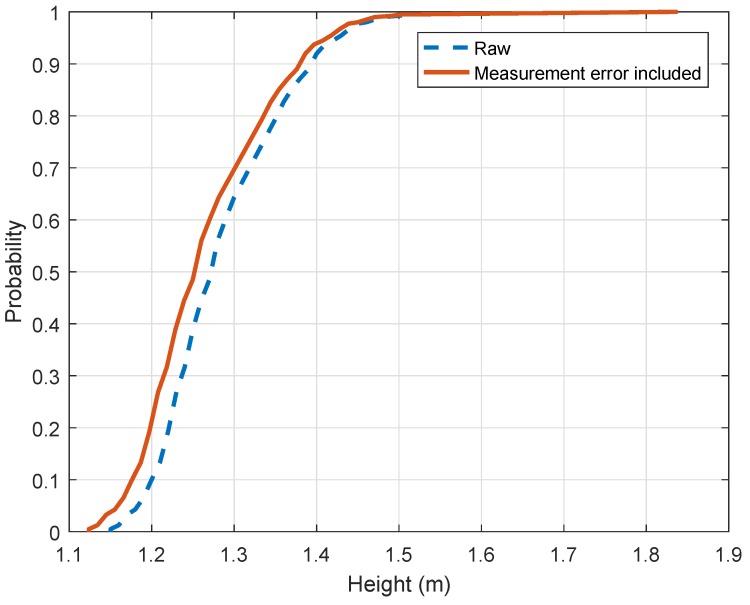
Empirical Cumulative Distribution of Heights of driver eye.

Height of driver’s height statistics were measured from the daytime dataset (c.f. Figure 1d and Figure 5a). They are based on 400 samples. Figure 8 details the heights extracted from our dataset. It displays the raw CDF as well as the corrected CDF given the measurement error obtained from the previous experiments. After correction, the median value is equal to 1.26 m (4.13 ft) while the mean value is 1.27 m (4.16 ft). The eye distribution is slightly biased toward the right. 1.18 m (3.87 ft) corresponds to the 10th percentile and 1.38 m (4.52 ft) corresponds to the 90th percentile. The 5th and 95th percentiles are respectively equal to 1.16 m (3.8 ft) and 1.42 m (4.65 ft). Values are larger than figures found in the literature. The second percentile is equal to 1.14 m in our study. The gap between the past and actual values is larger than the measurement uncertainty. Consequently, the drivers’ height standard should be updated. It might be explained by the increase of compact and large SUV shares in national automobile fleet from 2010s.

### 3.6. Height of the Rear Taillight Lower Limit

**Figure 9:**
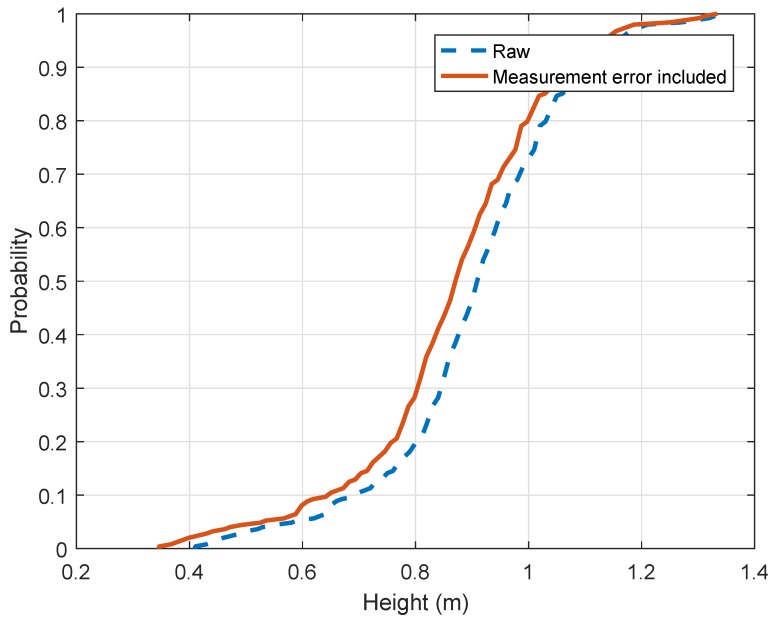
Empirical Cumulative Distribution of Height of the rear taillight lower limit.

The dataset used to obtain the rear taillight heights is the low light dataset. In fact, taillights are easier to select when the lights are on. It includes 250 vehicles Figure 9 shows the raw CDF as well as the corrected CDF given the measurement error obtained. After correction, the median value of taillights is equal to 0.87 m (2.85 ft) while the mean value is also 0.87 m (2.85 ft). The taillight distribution does not seem biased. 0.65 m (2.67 ft) corresponds to the 10th percentile and 1.07 m (3.51 ft) corresponds to the 90th percentile. The 5th and 95th percentiles are respectively equal to 0.53 m (1.73 ft) and 1.14 m (3.74 ft). It can be seen that the taillight height follows the same trend as the height of driver’s eye. It might be explained by higher vehicles on the road or taillights located higher on the vehicles.

## 4. Conclusions

This paper presents a framework to achieve height measurements from roadside ground level cameras. Our method automatically estimates the camera pose with respect to the road. It then requires selecting two points to obtain the height. Our framework does not require the traffic to be stopped. It offers flexibility regarding the camera setup installation. Our results suggest that the measurement error is bounded by 3 cm (1.18 in) despite our low-resolution camera. One could expect more accurate results with higher resolution cameras. The magnitude is as large as the ground truth uncertainty. Our results suggest that heights of driver’s eyes have increased since 2010. The trend is similar for taillight heights. In fact, the gap between current standards and our findings is larger than the measurement uncertainty. Our future work will probably be focused on robust automatic detection of tire-ground contact points for large-scale height measurements. We will also perform statistical analysis from the 3D information that could be extracted, such as the lateral position of the vehicle. Tests will also be performed in different environments. A study of eye and taillight height will also be carried out for different categories of vehicles (van, trailers, recreational vehicles, camper trailers,...)

## Figures and Tables

**Table 1:** Vehicle height measurement performance. MAE: Mean Absolute Error, RMSE: Root Mean Squared Error, MSE: Mean Squared Error, SD: Standard Deviation.

	Daytime	Low Light	Overall
MAE	0.023 m (0.075 ft)	0.0147 m (0.048 ft)	0.0203 m (0.066 ft)
RMSE	0.028 m (0.092 ft)	0.019 m (0.062 ft)	0.026 m (0.085 ft)
MSE	8 × 1e-4 m (2.6 × 1e-3 ft)	3.5 × 1e-4 m (1.1 × 1e-3 ft)	6.6 × 1e-4 m (2.1 × 1e-4 ft)
SD	0.026 m (0.085 ft)	0.019 (0.059 ft)	0.025 m (0.082 ft)
